# Agreement between laboratory-based and non-laboratory-based Framingham risk score in Southern Iran

**DOI:** 10.1038/s41598-021-90188-5

**Published:** 2021-05-24

**Authors:** Fatemeh Rezaei, Mozhgan Seif, Abdullah Gandomkar, Mohammad Reza Fattahi, Jafar Hasanzadeh

**Affiliations:** 1 Student Research Committee, Shiraz University of Medical Sciences, Shiraz, Iran; 2 Department of Epidemiology, School of Health, Shiraz University of Medical Sciences, Shiraz, Iran; 3 Non-Communicable Disease Research Center, Shiraz University of Medical Sciences, Shiraz, Iran; 4 Gastroenterohepatology Research Center, Shiraz University of Medical Sciences, Shiraz, Iran; 5 Research Centre for Health Sciences, Institute of Health, School of Health, Department of Epidemiology, Shiraz University of Medical Sciences, Shiraz, Iran

**Keywords:** Diseases, Health care, Risk factors

## Abstract

The Framingham 10-year cardiovascular disease risk is measured by laboratory-based and non-laboratory-based models. This study aimed to determine the agreement between these two models in a large population in Southern Iran. In this study, the baseline data of 8138 individuals participated in the Pars cohort study were used. The participants had no history of cardiovascular disease or stroke. For the laboratory-based risk model, scores were determined based on age, sex, current smoking, diabetes, systolic blood pressure (SBP) and treatment status, total cholesterol, and High-Density Lipoprotein. For the non-laboratory-based risk model, scores were determined based on age, sex, current smoking, diabetes, SBP and treatment status, and Body Mass Index. The agreement between these two models was determined by Bland Altman plots for agreement between the scores and kappa statistic for agreement across the risk groups. Bland Altman plots showed that the limits of agreement were reasonable for females < 60 years old (95% CI: −2.27–4.61%), but of concern for those ≥ 60 years old (95% CI: −3.45–9.67%), males < 60 years old (95% CI: −2.05–8.91%), and males ≥ 60 years old (95% CI: −3.01–15.23%). The limits of agreement were wider for males ≥ 60 years old in comparison to other age groups. According to the risk groups, the agreement was better in females than in males, which was moderate for females < 60 years old (kappa = 0.57) and those ≥ 60 years old (kappa = 0.51). The agreement was fair for the males < 60 years old (kappa = 0.39) and slight for those ≥ 60 years old (Kappa = 0.14). The results showed that in overall participants, the agreement between the two risk scores was moderate according to risk grouping. Therefore, our results suggest that the non-laboratory-based risk model can be used in resource-limited settings where individuals cannot afford laboratory tests and extensive laboratories are not available.

## Introduction

Cardiovascular Diseases (CVDs) are one of the leading causes of death globally^1^. The annual mortality of CVDs has been predicted to increase from 17.5 million in 2012 to 22.2 million by 2030. Then, CVDs would be the number one cause of death and disability worldwide^2^. In Iran, the share of non-communicable diseases in mortality increased from 57% in 1990 to 76% in 2010^3,4^ mostly due to ischemic heart disease, stroke, and other vascular diseases, which are generally considered CVDs^5^.

Most of the deaths related to CVDs are premature and preventable and can be improved using health management, effective diets, lifestyle interventions, and medication interventions^6^. A useful method to prevent CVDs is to assess the risk of CVDs regularly and to modify the lifestyle or the clinical treatment accordingly. Since the treatment of these diseases imposes heavy burdens and high costs on healthcare systems^7^, their early diagnosis and appropriate treatment have significant health benefits for patients with the highest absolute risk of CVDs^8^. Hence, the early prevention of CVDs can be considered the most useful and cost-effective intervention strategy. To effectively implement prevention strategies, reliable tools are required to identify individuals without overt CVDs who are at high risk for CVDs consequences in future. One of the tools utilized for predicting CVDs risk is the Framingham risk score, which can estimate the 10-year risk of fatal and non-fatal cardiovascular consequences.

In 2008, the Framingham risk scores developed by D’Agostino et al. that are calculated using either a laboratory-based or a non-laboratory-based algorithm. The laboratory-based model is based on age, sex, SBP and treatment status, current smoking, diabetes, cholesterol, and HDL. However, in developing countries, laboratory markers may not always be available at primary healthcare centers or people may not be able to pay for the cost of laboratory tests. In such cases, the non-laboratory-based algorithm can be used. The non-laboratory-based model is based on age, sex, SBP and treatment status, current smoking, diabetes and, BMI^9^.

The Framingham model, on the other hand, is a complex mathematical equation. Thus, the risk calculation formula has been simplified to a points-based system to make it easier to be used in places where there is no computer or calculator^10^. Determining the risk score using the points-based risk scoring method is easier than the equation-based method and is more applicable in places where primary healthcare is provided.

Given that CVDs are the most important cause of death in the Iranian population and their burden has been expected to increase in the coming years^11^, it seems necessary to estimate the 10-year risk of CVDs based on the risk algorithms. There is currently no specific CVD risk prediction tool developed in Iran. Therefore, we use created tools in other countries such as Framingham risk score and Systematic Coronary Risk Evaluation (SCORE) models. Some studies have compared different risk prediction models. In studies carried out in Sri Lanka, Rwanda, and Canada, the agreement between BMI-based and cholesterol-based models was examined^12–14^. In Iran, Bavarsad et al. compared Framingham and World health organization/International society of hypertension (WHO/ISH), and SCORE models^15^. Mirzaei et al. assessed Framingham and WHO/ISH models^16^. But no study has been done to examine the agreement between laboratory-based and non-laboratory-based Framingham risk scores.

The important issue to be examined is whether the two laboratory-based and non-laboratory-based risk models provide a similar estimate of the CVDs risk in an individual according to the points-based risk scoring system. Therefore, the present study aims to evaluate the agreement between laboratory-based and non-laboratory-based risk scores in a large population.

## Methods

### Pars cohort study design

This cross-sectional study was conducted using the baseline data of Pars cohort study. Pars cohort study is a part of the Persian Prospective Epidemiological Research Studies in Iranian Adults (PERSIAN) program. PERSIAN cohort study was designed and implemented in 2014 and included 18 different geographical, ethnic, and climatic groups in 18 provinces of Iran^17^.

Details of the Pars cohort study have already been published^18^. Briefly, it was conducted on 9,264 individuals aged 40–75 years living in Valashahr and neighboring villages (southern Iran). In Pars cohort study, trained interviewers collected information about demographic characteristics, lifestyle, and health history of the participants using structured questionnaires. They also measured their height, weight, and blood pressure. Blood samples were also taken for biochemical tests. In the present study, people with a history of CVDs or strokes were excluded. The sample contained 8138 cases, and all methods were performed in accordance with PERSIAN instructions.

### CVDs risk

In this study, the 10-year risk of CVDs was calculated using the Framingham risk scores of laboratory-based and non-laboratory-based models. For the laboratory-based model, scores were calculated using age, sex (male/female), systolic blood pressure (mmHg) and treatment status, current smoking status (yes/no), diabetes (yes/no), and total cholesterol and HDL (mg/dL). For the non-laboratory-based model, scores were calculated using age, sex (male/female), systolic blood pressure (mmHg) and treatment status, current smoking status (yes/no), diabetes (yes/no), and BMI (kg/m^2^)^9^.

This study considered a smoker as someone who had smoked ≥ 100 cigarettes, and a current smoker as someone who smoked regularly. Diabetes status was assessed by the previous history of the disease. Blood pressure was measured by a trained individual using a mercury sphygmomanometer after five minutes of sitting. Blood pressure was measured twice with a 10-min interval from each arm and the mean blood pressure was recorded. Cholesterol and HDL were tested in the laboratory. Finally, BMI was evaluated by dividing weight by height squared (kg/m^2^).

### Statistical analysis

Percentage was reported for grouped data and mean and standard deviation for quantitative data. Chi-square and t-test were used for categorical and continuous variables, respectively. The risk of CVDs was calculated for laboratory-based and non-laboratory-based models using the points‐based risk‐scoring system. Two methods were used to determine the agreement between the two models. In the first method, the risk score of CVDs was considered a quantitative variable and the agreement between the two models was examined using Bland Altman plots. It means that the risk difference between the non-laboratory-based and laboratory-based models was calculated after determining the risk^14^. The difference between the mean risk scores was also calculated by gender and age groups (< 60 and ≥ 60 years). In Bland Altman plots, the difference between the two scores was shown on the vertical axis and the mean of the two scores on the horizontal axis. Since the true risk of CVDs is uncertain for each individual, the mean of laboratory-based and non-laboratory-based scores is the best estimate available^14^. The mean difference of the scores + /− two standard deviations represent 95% of the limit of agreement. The interval made by 95% of the limit of agreement indicates that 95% of the difference between the two scores is not real^19^.

In the second method, the risk of CVDs was categorized. In both models, individuals were divided into low-risk (< 10%), moderate-risk (10–20%), and high-risk (> 20%) groups. Then, the agreement between the laboratory-based and non-laboratory-based risk models was determined using kappa statistics. Kappa statistics < 0 indicated the agreement less than odds and values 0.01–0.20, 0.21–0.40, 0.41–0.60, 0.61–0.80, and 0.81–0.99 represented slight, fair, moderate, substantial, and almost complete agreement, respectively^20^.

### Ethical considerations

This study was approved by the Ethics Committee of Shiraz University of Medical Sciences (IR.SUMS.REC.1398.860). The data were collected anonymously and informed consent forms were obtained from the participants.

## Results

Among the 8138 participants in this study, 3789 (46.56%) were male. The mean age of the participants was 51.65 ± 9.06 years. In addition, 14.41% were smokers. The prevalence of smoking was significantly higher in males (30.22% vs. 0.64% in females). The prevalence of hypertension was 12.79%. Hypertension was significantly more prevalent in females (17.68%vs. 7.18% in males)**.** Besides, the prevalence of diabetes was 8.38%. Diabetes was significantly more prevalent in females (10.99% vs. 5.38% in males). Abdominal obesity was significantly higher in females (89.33%vs. 70.60% in males).

The mean diastolic blood pressure was significantly higher in males than in females (73.44 ± 11.61 vs. 72.92 ± 11.91). However, the mean systolic blood pressure was higher in females than in males (111.54 ± 19.64 vs. 110.78 ± 17.50). The means of HDL and cholesterol were also significantly higher in females compared to males. BMI was also significantly higher in females than in males (27.05 ± 4.73 vs. 24.31 ± 4.08).

The mean of the 10-year risk of CVDs in the general research population was higher in the non-laboratory-based model than in the laboratory-based model (9.38 ± 7.38 vs. 6.68 ± 6.15). In addition, the mean of the 10-year risk of CVDs was significantly higher in males compared to females in both models (Table 1).

**Table 1:** Reporting of the participants’ characteristics.

Variables	Total (n = 8138)	Males (n = 3789)	Females (n = 4349)	*P* value
	N (%)	N (%)	N (%)	
**Age range (years)**				
< 60	6457 (79.34)	3023 (79.78)	3434 (78.96)	0.36*
≥ 60	1681 (20.66)	766 (20.22)	915 (21.04)	
**Marital status**				
Married	7257(89.17)	3696(97.55)	3561(81.88)	< 0.001*
Other	881(10.83)	93(2.45)	788(18.12)	
**Education level**				
Illiterate	3795 (46.63)	1107 (29.22)	2688 (61.81)	< 0.001*
≤ diploma	4081 (50.15)	2438 (64.34)	1643 (37.78)	
University	262 (3.22)	244 (6.44)	18 (0.41)	
**Smoking (now)**				
No	6965 (85.59)	2644 (69.78)	(99.36) 4321	< 0.001*
Yes	1173 (14.41)	1145 (30.22)	28 (0.64)	
**Hypertension**				
No	7097 (87.21)	3517 (92.82)	3580 (82.32)	< 0.001*
Yes	1041 (12.79)	272 (7.18)	769 (17.68)	
**Diabetes**				
No	7456 (91.62)	3585 (94.62)	3871 (89.01)	< 0.001*
Yes	682 (8.38)	204 (5.38)	478 (10.99)	
**Abdominal obesity**				
No	1578 (19.39)	1114 (29.40)	464 (10.67)	< 0.001*
Yes	6560 (80.61)	2675 (70.60)	3885 (89.33)	
DBP (Mean mmHg ± SD)	73.16 ± 11.77	73.44 ± 11.61	72.92 ± 11.91	0.04**
SBP (Mean mmHg ± SD)	111.19 ± 18.68	110.78 ± 17.50	111.54 ± 19.64	0.06**
HDL (Mean mmol/l ± SD)	1.49 ± 0.33	1.40 ± 0.30	1.56 ± 0.34	< 0.001**
Chol (Mean mmol/l ± SD)	5.06 ± 1.06	4.85 ± 1	5.24 ± 1.08	< 0.001**
BMI (kg/m^2^)	25.77 ± 4.64	24.31 ± 4.08	27.05 ± 4.73	< 0.001**
Laboratory-based CVDs risk score (10- year,%), (Mean ± SD)	6.68 ± 6.15	9.04 ± 6.74	4.62 ± 4.69	< 0.001**
Non-laboratory-based CVD risk** score** (10- year,%), (Mean ± SD)	9.38 ± 7.63	13.02 ± 7.89	6.20 ± 5.74	< 0.001**

*DBP* diastolic blood pressure, *SBP* systolic blood pressure, *HDL* high density lipoprotein, *Chol*: cholesterol.

*chi-square test

**t-test

### Mean differences in risk scores

Among all participants, the mean difference between the laboratory-based and non-laboratory-based scores was 2.69% (95% CI: 2.63% to 2.76%), which was 3.97% (95% CI: 3.86% to 4.08%) for males and 1.58% (95% CI: 1.51% to 1.64%) for females. The mean difference in scores was 2.23% (95% CI: 2.16% to 2.29%) among the participants < 60 years old and 4.48% (95% CI: 4.28% to 4.68%) among those ≥ 60 years old.

The mean difference of the scores was also calculated based on the age groups. Accordingly, the mean difference of the scores was 3.43% (95% CI: 3.33% to 3.52%) in the males < 60 years old, 6.11% (95% CI: 5.79% to 6.44%) among the males ≥ 60 years old, 1.17% (95% CI: 1.11% to 1.23%) in the females < 60 years old, and 3.11% (95% CI: 2.90% to 3.32%) among the females ≥ 60 years old.

### Bland–Altman plots/limits of agreement

**Figure 1:**
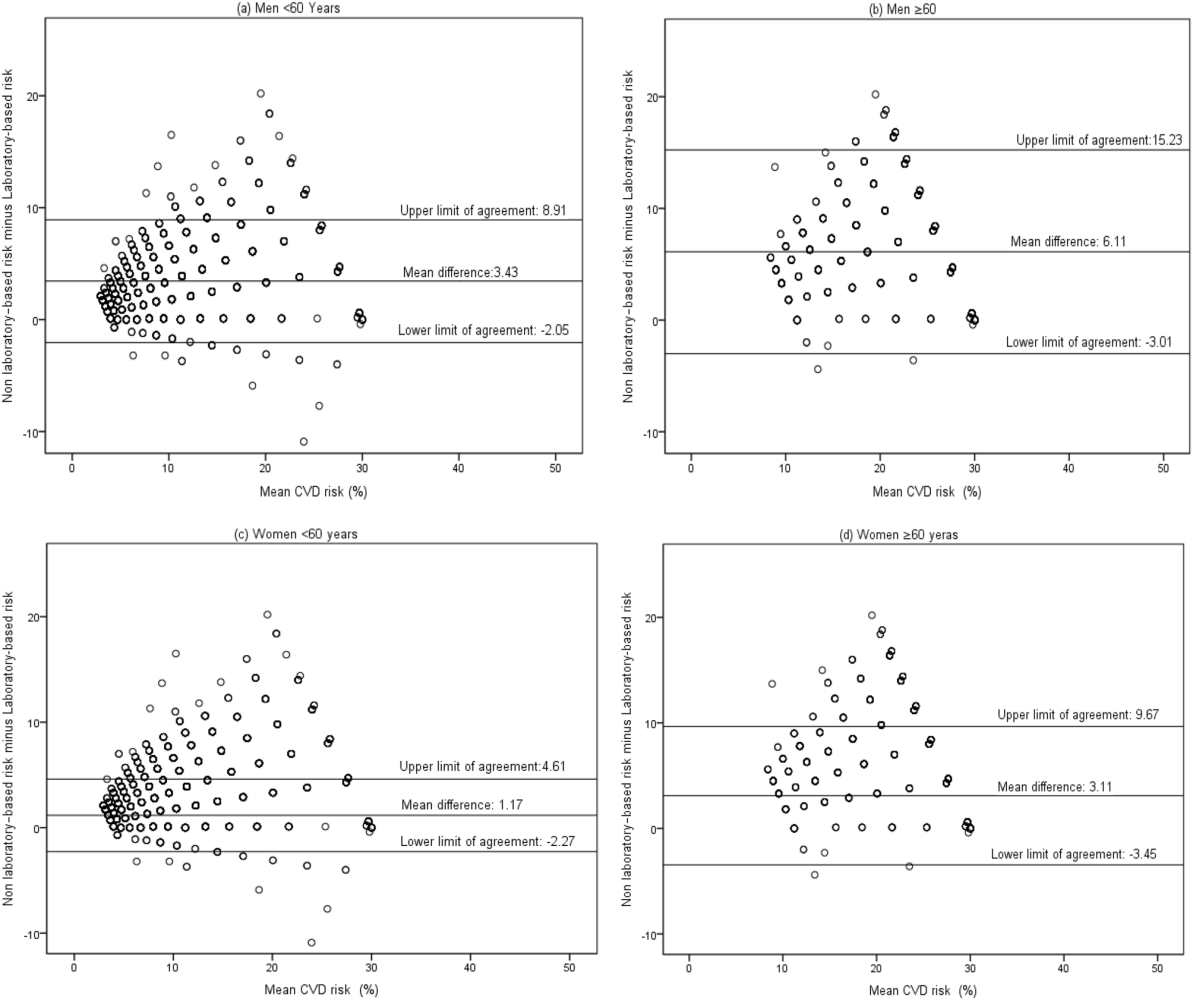
Bland–Altman plots showing agreement between the laboratory-based and non-laboratory-based CVDs risk scores.

Bland Altman plots of the agreement between the two risk scores for males and females < 60 and ≥ 60 years old have been presented in Fig. 1. Accordingly, the limit of agreement was better for females < 60 years old compared to females ≥ 60 years old, males < 60 years old, and males ≥ 60 years old. The limit of agreement was −2.27% to 4.61% for females < 60 years old, 9.67% to -3.45% for females ≥ 60 years old, -2.5% to 8.91% for males < 60 years old, and -3.01% to 15.23% for males ≥ 60 years old. The limit of agreement was wider for males ≥ 60 years old in comparison to other groups.

### Categorical agreement

In the total research population, the agreement of the two risk scores was 78.79%. The agreement between the two risk scores according to the risk score categories in males and females has been presented in Tables 2 and 3, respectively. For males, the agreement between the risk scores was equal to 66.43% (kappa = 0.42, Standard Error (SE) = 0.01). It should be noted that there was a larger number of participants in the high-risk group for the non-laboratory-based model than in the laboratory-based model (733 vs. 320). Disagreement was observed in both low-risk and high-risk groups. Non-laboratory-based risk groups tended to show a higher risk, such a way that 1238 males (32.67%) were in the risk groups higher than the laboratory-based risk, but only 12 (0.32%) were in the lower risk group.

**Table 2:** Agreement between the laboratory-based and non-laboratory-based risk scores according to the grouped risk in males.

Non-laboratory-based risk category	Laboratory-based risk category				Agreement (%)	Kappa
	Low	Moderate	High	Total		
**All males**						
Low	1790	8	0	1798	66.43	0.42
Moderate	843	411	4	1258		
High	22	395	316	733		
Total	2655	814	320	3789		
** < 60 years old**						
Low	1790	8	0	1798	71.95	0.39
Moderate	672	300	4	976		
High	7	157	85	249		
Total	2469	465	89	3023		
**≥ 60 years old**						
Low	0	0	0	0	44.65	0.14
Moderate	171	111	0	282		
High	15	238	231	484		
Total	186	349	231	766

**Table 3:** Agreement between the laboratory-based and non-laboratory-based risk scores according to the grouped risk in females.

Non-laboratory-based risk category	Laboratory-based risk category				Agreement (%)	Kappa
	Low	Moderate	High	Total		
**All females**						
Low	3551	13	0	3564	89.56	0.59
Moderate	314	257	7	578		
High	9	111	87	207		
Total	3874	381	94	4349		
** < 60 years old**						
Low	3106	10	0	3116	94.26	0.57
Moderate	151	118	4	273		
High	2	30	13	45		
Total	3259	158	17	3434		
**≥ 60 years old**						
Low	445	3	0	448	71.91	0.51
Moderate	163	139	3	305		
High	7	81	74	162		
Total	615	223	77	915		

For males < 60 years old, the agreement was 71.95% (kappa = 0.39, SE = 0.01). For this age group, the disagreement was observed in both directions. In the non-laboratory-based model, there was a larger number of participants in the high-risk group compared to the laboratory-based model (249 vs. 89). In the non-laboratory-risk categories, there was a larger number of participants in the higher risk groups than in the laboratory-based risk categories [829 (27.42%)], but only 12 males (0.40%) were in the lower risk groups.

For males ≥ 60 years old, the agreement was 44.65% (kappa = 0.14, SE = 0.02). In this age group, there were more participants in the high-risk group in the non-laboratory-based model than in the laboratory-based model (484 vs. 231). In the non-laboratory-based risk model, there was no male participant in the low-risk group, while there were 186 males (24.28%) in the low-risk group in the laboratory-based model. In the non-laboratory-based risk group, there was a larger number of participants in the higher risk groups compared to the laboratory-based risk categories [409 (53.39%)], but there were no males in the lower risk groups.

For all females, the agreement was 89.56% (kappa = 0.59, SE = 0.01). In the non-laboratory-based risk model, there was a larger number of females in the high-risk group in comparison to the laboratory-based risk model (207 vs. 94). The disagreement was observed in both low-risk and high-risk groups. Yet, non-laboratory-based risk categories were more likely to show a higher risk compared to the laboratory-based risk categories. Accordingly, there were 425 females (9.77%) in the higher risk groups, but only 20 (0.46%) in the lower risk groups.

Considering the females < 60 years old, the agreement was 94.26% (kappa = 0.57, SE = 0.02). For this age group, the disagreement was observed in both low-risk and high-risk groups. In the non-laboratory-based risk model, there was a larger number of participants in the high-risk group compared to the laboratory-based risk model (45 vs. 17). In the non-laboratory-risk categories, there were more females in the higher risk groups (181, 5.27%) in comparison to the laboratory-based risk categories, but there were only 14 females (0.41%) in the lower risk groups.

For females ≥ 60 years old, the agreement was 71.91% (kappa = 0.51, SE = 0.02). In the non-laboratory-based risk model, there was a larger number of females in the high-risk group (162 vs. 77) in comparison to the laboratory-based risk model. In the non-laboratory-based risk categories, there were more females in the higher risk groups (244, 26.67%) compared to the laboratory-based risk categories, but there were only six females (0.65%) in the lower risk groups.

## Discussion

In the present study, the agreement between Framingham 10-year general CVDs risk was measured by two laboratory-based and non-laboratory-based models using Bland Altman plots and kappa statistics in a large population using the points-based risk scoring system. Since no calculator or computer program may be available to calculate the Framingham risk according to the risk equation in the centers where primary healthcare is provided, the staff of these centers calculate the risk of CVDs using the points-based risk scoring system. On the other hand, due to the high cost and lack of resources, it is not always possible for patients to perform laboratory tests and in some settings, the non-laboratory-based method is used to determine the risk of CVDs.

Up to now, few studies have measured the agreement between laboratory-based and non-laboratory-based CVDs risk scores using the risk scoring equation-based system that uses Cox proportional hazards regression. In the current study, Bland–Altman plots showed that the agreement between the two risk scores was better among the females aged < 60 years and was less among the males ≥ 60 years old. Moreover, the limit of agreement was appropriate for the females < 60 years old (95% CI: -2.27% to 4.61%), but not for other groups (95% CI: -3.45% to 9.67% for females ≥ 60 years old and 95% CI: -2.05% to 8.91% for males < 60 years old). For males ≥ 60 years old, the limit of agreement was wider in comparison to other age groups (95% CI: -3.01% to 15.23%). In a study carried out on South Asian Canadians, the agreement between BMI-based and cholesterol-based models was examined with an equation-based risk scoring system. The results were similar to those of the present study and showed that the difference between the two scores and the change in the differences increased by increase in the mean 10-year risk of CVDs. In terms of clinical significance, the limit of agreement was appropriate for the females < 60 years old^14^. In this regard, the current study found that the limit of agreement was appropriate for males and females < 60 years old, but not for the other participants.

In this study, the mean difference between the risk scores of laboratory-based and non-laboratory-based models in the total population was 2.69%, which was higher in males than in females (3.97% vs. 1.58%). However, according to grouping, the mean difference in scores was higher in older females and males (3.43% in males < 60 years old and 6.11% in males ≥ 60 years old). Moreover, the mean difference in scores was less in females than in males (1.17% in females < 60 years old and 3.11% in those ≥ 60 years old). In another study, the mean difference in the scores of the two models was lower compared to the present study (0.5% among males and 0.6% among females)^14^.

In the present study, the kappa statistics for the risk categories in the two models indicated that the agreement between the two risk scores was better in females than in males. The agreement was moderate in all females although the kappa statistic was higher in the females < 60 years old compared to those aged ≥ 60 years, but both age groups had a moderate agreement. The agreement was also moderate for all males. Nonetheless, when kappa statistics were measured by age groups, the agreement was fair for the males < 60 years old and slight for those aged ≥ 60 years. Pandya et al. revealed a high agreement between the laboratory-based and non-laboratory-based risk scores, and stated that the non-laboratory-based score could be a good proxy of Framingham risk scores in case resources were limited^21^.

Gaziano et al. also showed that the non-laboratory-based model correctly predicted the fatal and non-fatal consequences of CVDs in the laboratory-based model^22^. In these studies, the limit of agreement was not examined at the individual level. However, the agreement between the two models in each individual was assessed using Bland Altman plots in the present study. The results of a longitudinal study in Iran demonstrated that the Framingham risk of the non-laboratory-based model was comparable to the laboratory-based model. However, that study used the Cox proportional hazards model to determine the risk^23^, while the present study used the points-based risk scoring system. The results of another study in Iran showed that the agreement between the CVDs risk groupings was negligible with different risk prediction models^16^.

The results of the present study showed that the non-laboratory-based risk categories tended to show a higher risk compared to the laboratory-based risk model. Accordingly, in the non-laboratory-based model, the number of males and females in the high-risk group were slightly more than twice as the laboratory-based model. Probably, the method of measuring BMI could underestimate or overestimate the risk of CVDs, which might increase or decrease the laboratory-based and non-laboratory-based risk scores^14^. Even if we assume that there was a BMI measurement error, this error had randomly occurred in the whole research population. However, the Pars cohort study used accurate measurement tools, which could guarantee the accuracy of the data. Yet, it should be considered that obese people have other risk factors, because obesity has an important role in hypertension, hyperlipidemia, and hyperglycemia and is independently associated with a higher cardiovascular risk^24–26^. Therefore, the non-laboratory-based model showed a higher risk of CVDs in comparison to the laboratory-based model. Similarly, the results of the study by Jones et al. indicated that the BMI-based risk model tended to show higher risks compared to the cholesterol-based risk model. Nonetheless, in that study, the agreement was moderate for all groups, except for males aged 60–74 years, which showed a fair agreement^14^. In the RODAM study, the agreement between laboratory-based and non-laboratory-based Framingham risk scores was 74.8%, with a Kappa statistic of 0.63^27^, which was almost similar to the general agreement in the present study. The results of the research by Gray et al. indicated that the non-laboratory algorithm predicted a higher absolute risk in comparison to the laboratory algorithm^28^. However, due to ethnic, racial, and geographical differences, risk instruments created in one population might not accurately predict the real risk in other populations. Therefore, it seems necessary to validate risk scores in different populations.

The present study results demonstrated that the non-laboratory-based model could not be used instead of the laboratory-based model. However, CVDs risk assessment using the non-laboratory-based model has been shown to have the potential to improve the usefulness of risk scores and CVDs prevention efforts worldwide^29,30^. Therefore, especially in settings with limited resources where extensive laboratories are not available and it is not economically possible to perform laboratory tests^31^, the non-laboratory-based model is recommended because it tends to show a higher risk of CVDs and is more conservative compared to the laboratory-based model.

### Study strengths and limitations

The present study is the first population-based study to examine the agreement between Framingham laboratory-based and non-laboratory-based CVDs risk scoring methods in a large Iranian population. Due to the large sample size, the findings of this study can be generalized. The accuracy of the data can also be guaranteed because data collection was performed using accurate and reliable tools. However, the present study followed a cross-sectional design based on the baseline data of a cohort study. Thus, a longitudinal study with an adequate follow-up period is required to validate the laboratory-based and non-laboratory-based risk models in the study population. Also, because there is no specific CVD prediction risk tool in Iran. So, we used the Framingham risk score that was developed for the Caucasian population. It seems that modification of the Framingham risk score for the Iranian population is essential.

## Conclusion and recommendations

In the general population, the agreement between the two risk scores was moderate according to risk grouping. The non-laboratory-based model is measured without a blood test. Risk factors information of this model can be obtained easily and quickly. Therefore, the non-laboratory-based risk model can be used in resource-limited settings where individuals cannot afford laboratory tests. Future longitudinal cohort studies are suggested to measure the discrimination of these two risk scores in the covered population with an adequate follow-up period. Also, future studies are recommended to modify the Framingham risk score for the Iranian population.
